# Delayed diagnosis of the full triad autoimmune polyendocrine syndrome type 2 with adrenal crisis: a case report and literature review

**DOI:** 10.3389/fimmu.2025.1563629

**Published:** 2025-05-09

**Authors:** Zihong Yao, Hui Chen, Xuejian Hu, Dan Ge, Xiangyu Xu, Danxia Xu

**Affiliations:** ^1^ The Second Clinical Medical College, Lanzhou University, Lanzhou, Gansu, China; ^2^ Department of Endocrinology and Metabolism, Lanzhou University Second Hospital, Lanzhou, Gansu, China

**Keywords:** autoimmune polyendocrine syndrome type 2, adrenal insufficiency, adrenal crisis, hypothyroidism, diabetes mellitus

## Abstract

**Background:**

Autoimmune polyendocrine syndrome type 2 (APS-2) is a rare disorder characterized by autoimmune damage to multiple endocrine glands and typically involves primary adrenal insufficiency (PAI), autoimmune thyroid disease (AITD), and type 1 diabetes mellitus (T1DM). Clinical presentations that feature the full triad alongside adrenal crisis (AC) are rare, with only four such cases reported globally. While AC is the most life-threatening acute complication of APS-2, its pathogenesis is complex and incompletely understood. While there are multiple potential triggers, the role of exogenous substances such as traditional Chinese medicine [TCM] has not been systematically examined.

**Case presentation:**

A 69-year-old female was hospitalized with a 9-year history of increasing fatigue, which had recently worsened due to high fever, anorexia, and vomiting lasting 2 days. She has previously been diagnosed with T1DM (nine years prior) and AITD (five years prior). Four years earlier, she underwent thymoma resection. Three years before admission, she self-administered an unknown TCM remedy that coincided with increased fatigue and mucocutaneous hyperpigmentation. On admission, she was in hypovolemic shock and severe hyponatremia (118.0 mmol/L). Laboratory tests revealed low basal cortisol (2.38 μg/dL) and markedly elevated adrenocorticotropic hormone (>1250 pg/mL). An adrenocorticotropic hormone stimulation test confirmed non-responsive adrenal function, indicating PAI. Together with her medical history and positive antibody profile, APS-2 with AC was diagnosed. She responded well to high-dose intravenous glucocorticoid therapy, sodium supplementation, and symptomatic management. Although persistent hyponatremia recurred following discharge, it resolved following fludrocortisone acetate supplementation, and her condition remained stable at the last follow-up.

**Conclusion:**

We report the fifth case of full-triad APS-2 with AC and document a 9-year diagnostic delay due to non-specific symptoms with asynchronous multi-glandular involvement. Thyroxine replacement therapy and potential TCM-induced changes may have aggravated cortisol metabolism and immune imbalances, hastening adrenal failure. Clinicians should implement stepwise organ-function monitoring in patients with any single-gland autoimmune disease, maintain vigilance for exogenous medication use, and implement multidisciplinary management strategies to mitigate the risk of AC. This case provides critical insights into both the pathogenesis and clinical management of APS-2.

## 1 Introduction

Autoimmune polyendocrine syndrome type 2 (APS-2) is a rare disorder characterized by progressive multi-glandular dysfunction, classically involving primary adrenal insufficiency (PAI), autoimmune thyroid disease (AITD), and type 1 diabetes mellitus (T1DM) (1). The full triad of APS-2 is exceptionally rare in clinical practice, and cases progressing to adrenal crisis (AC) are especially challenging; only four such cases have been previously reported worldwide (2–5). Although AC represents the most severe acute complication of APS-2, its pathogenesis remains poorly understood. Recognized triggers include infections, surgery, and medications (6, 7), but systematic research into multifactorial mechanisms, especially the potential adrenal toxic effects of exogenous agents such as traditional Chinese medicine (TCM), is lacking. Importantly, cases of delayed diagnosis, exceeding 60% in APS-2, are closely linked to disease heterogeneity, non-specific clinical manifestations, and limited clinician awareness, factors that exacerbate AC risk (8–10). Early diagnosis is critical for APS-2 patients, as it enables timely initiation of hormone replacement therapy to prevent life-threatening complications like AC and significantly reduces irreversible organ damage and mortality linked to delayed intervention. Enhancing clinician awareness of the disease’s heterogeneous presentations and implementing standardized screening protocols are key strategies to improve outcomes.

This report presents the fifth global case of full-triad APS-2 complicated by AC, emphasizing important clinical insights gained through a years-long diagnostic delay. This patient’s disease progression highlights a temporal association between TCM exposure and PAI deterioration, providing direct clinical evidence for multifactorial AC pathogenesis. Three critical knowledge gaps are highlighted: (1) limited recognition of the heterogeneous presentation of APS-2 by non-endocrinilogists; (2) insufficient attention to exogenous factors such as TCM in clinical practice; and (3) the need for systematic monitoring of adrenal cortex function in APS-2 patients to optimize replacement therapy. These findings offer valuable guidance for clinical management strategies and future research into TCM-induced adrenal toxicity.

## 2 Case presentation

A 69-year-old female was admitted to the endocrinology department in May 2023 due to progressive fatigue over nine years, acutely worsened by high fever, anorexia, and vomiting for two days. Her clinical history illustrates a typical progression of multiglandular failure. Nine years prior, she developed fatigue, poor appetite, dry mouth, polydipsia, and polyuria. Laboratory tests revealed elevated fasting blood glucose (FBG) of 22.0 mmol/L, positive insulin autoantibody (IAA), and anti-glutamic acid decarboxylase antibody (Anti-GAD antibody), confirming T1DM. Insulin therapy partially improved her symptoms. Five years prior, her fatigue worsened, associated with thyroid dysfunction: reduced free triiodothyronine (FT3) and free thyroxine (FT4), elevated thyroid-stimulating hormone (TSH), positive anti-thyroglobulin antibody (TGAb), and anti-thyroid peroxidase antibody (TPOAb). Diffuse thyroid lesions on ultrasound confirmed AITD with primary hypothyroidism, and levothyroxine replacement was initiated. Four years earlier, she developed bilateral ptosis and generalized myasthenia. Repetitive nerve stimulation indicated low-frequency decrement, neostigmine test was positive, and chest CT revealed an anterior mediastinal mass. Postoperative pathology confirmed thymoma (type B1). Perioperative glucocorticoids and pyridostigmine bromide were briefly used but discontinued post-discharge. No tumor recurrence was observed during outpatient follow-up. Three years prior, self-administration of an unspecified TCM worsened fatigue and caused patchy pigmentation on the tongue, later progressing to generalized mucocutaneous hyperpigmentation. Two days before admission, she experienced abrupt fatigue exacerbation, high fever, severe anorexia, as well as frequent nausea and vomiting, with a recent 4 kg weight loss. She had a 5-year history of orthostatic hypotension (blood pressure: 101/70 → 70/50 → 50/40 mmHg). No significant family history, postpartum hemorrhage, tuberculosis, or chronic kidney disease was reported.

**Figure 1:**
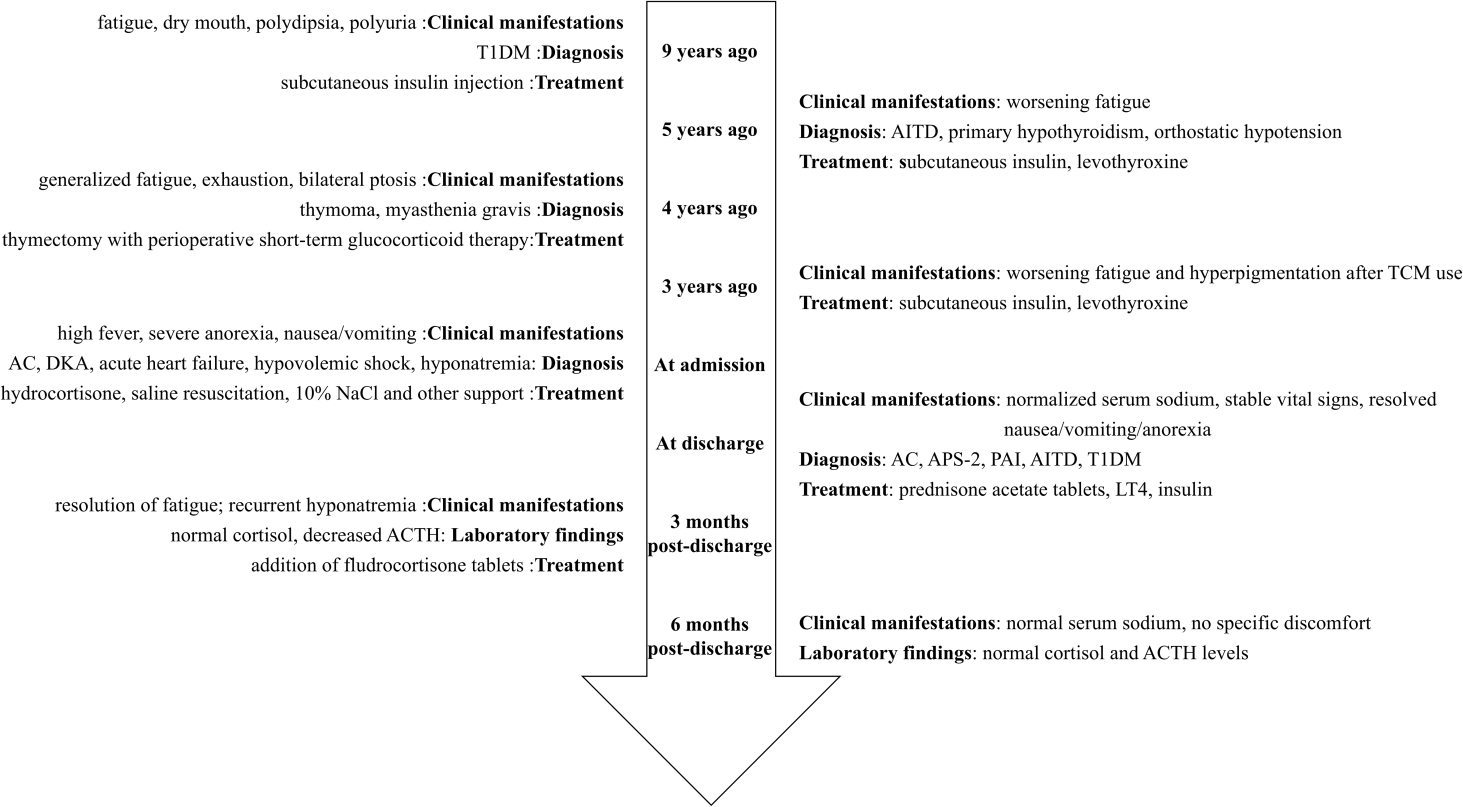
Timeline flowchart of disease progression and interventions. T1DM, Type 1 diabetes mellitus; AC, Adrenal crisis; DKA, Diabetic ketoacidosis; ACTH, Adrenocorticotropic hormone; AITD, Autoimmune thyroid disease; TCM, Traditional Chinese medicine; APS-2, Autoimmune polyendocrine syndrome type 2; PAI, Primary adrenal insufficiency.

On admission, the patient appeared lethargic, cachectic, with dry skin, sunken eyes, cold extremities, and extensive hyperpigmentation predominantly affecting non-sun-exposed areas, including the cheeks, nasolabial folds, tongue, palms, soles, elbows, and buttocks (**Figure 1**). Vital signs showed a blood pressure of 75/47 mmHg, heart rate of 140 bpm, BMI of 21.3 kg/m², and temperature of 39.0°C. Laboratory tests revealed severe hyponatremia (118.0 mmol/L), hypochloremia (87.1 mmol/L), elevated FBG (20.5 mmol/L), HbA1c (9.2%), and urine ketones (2+). Markers of cardiac stress were elevated, including NT-proBNP (22,863.00 pg/mL) and hs-cTnT (33.05 ng/L). IAA, GAD antibody, and ANA were positive. Complement components (C3, C4), serum creatinine, eGFR, and urine protein levels were normal. Thyroid ultrasound revealed bilateral hyperechoic nodules (C-TIRADS 3) with diffuse parenchymal changes.

Given her chronic fatigue, orthostatic hypotension, acute-onset nausea and vomiting, anorexia, mucocutaneous hyperpigmentation, and hypovolemic shock, adrenal function tests were performed. Laboratory findings revealed a low cortisol level with markedly elevated ACTH. An ACTH stimulation test confirmed adrenal insufficiency, with a 60-minute cortisol level of 1.8 μg/dL, well below the stimulation threshold. Adrenal CT revealed normal morphology without masses or calcification. Electrocardiography, pituitary hormone levels, and pituitary CT were unremarkable. Echocardiography indicated normal systolic function with a left ventricular ejection fraction of 63%.

The final diagnoses included AC, APS-2, PAI, AITD (on thyroid replacement), T1DM, diabetic ketoacidosis (DKA), acute heart failure, severe hyponatremia, and hypochloremia. Immediate management included intravenous hydrocortisone (100 mg Q8h), saline resuscitation, 10% sodium chloride infusion, insulin therapy, and cardiac support. Within 24 hours, her blood pressure improved to 106/63 mmHg, and gastrointestinal symptoms were resolved. Hyponatremia was corrected to 136.8 mmol/L, and urine ketones became negative within 72 hours. Intravenous hydrocortisone was gradually tapered and transitioned to oral prednisone. At discharge, the patient’s electrolytes had normalized, hyperpigmentation had significantly decreased, and symptoms such as fatigue and anorexia had resolved.

**Figure 2:**
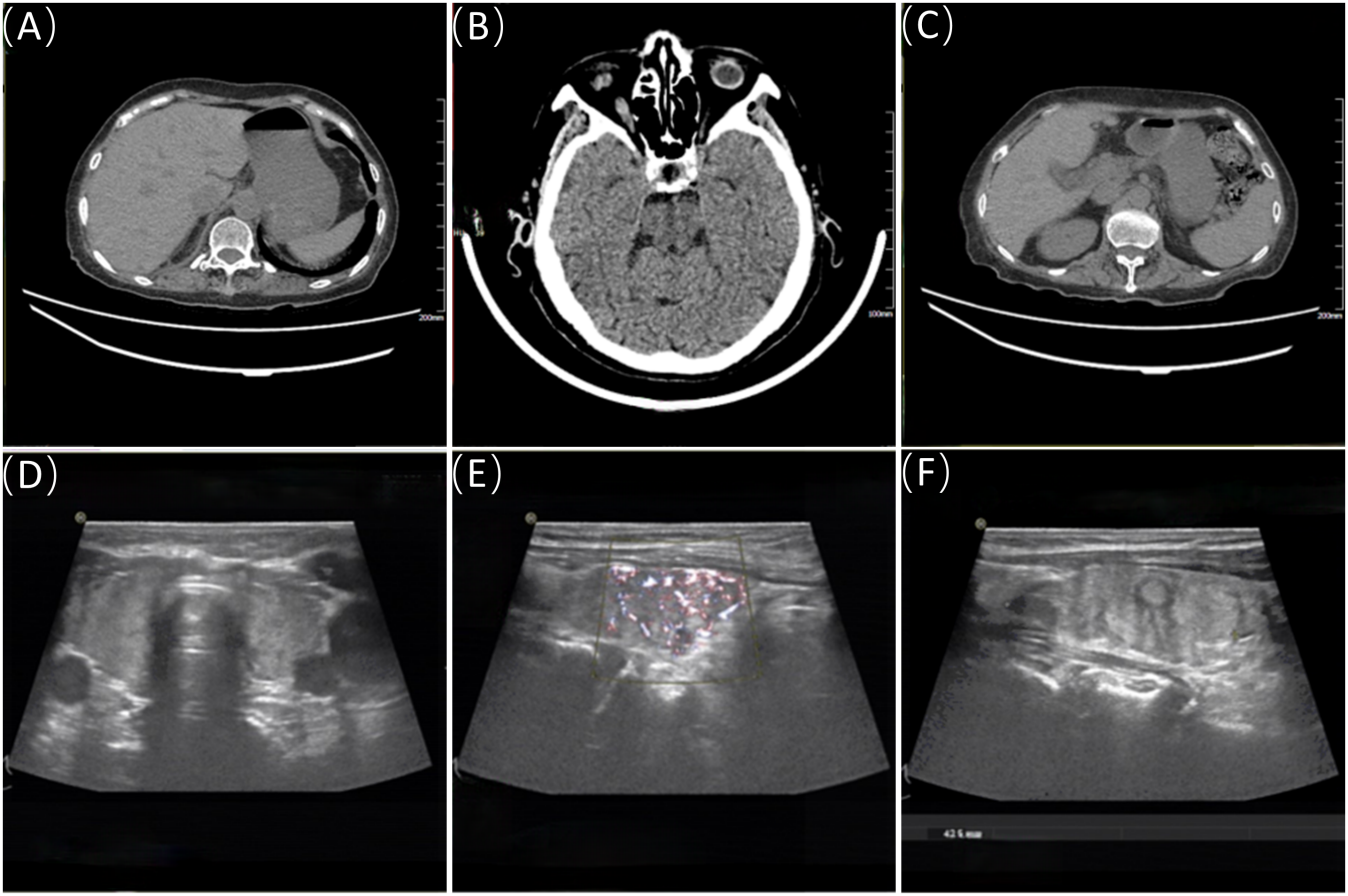
Adrenal CT, pituitary CT, and thyroid ultrasound. **(A, C)** show the CT images of the right and left adrenal glands, respectively; **(B)** displays the pituitary CT image. **(D–F)** are ultrasound images of the thyroid.

**Figure 3:**
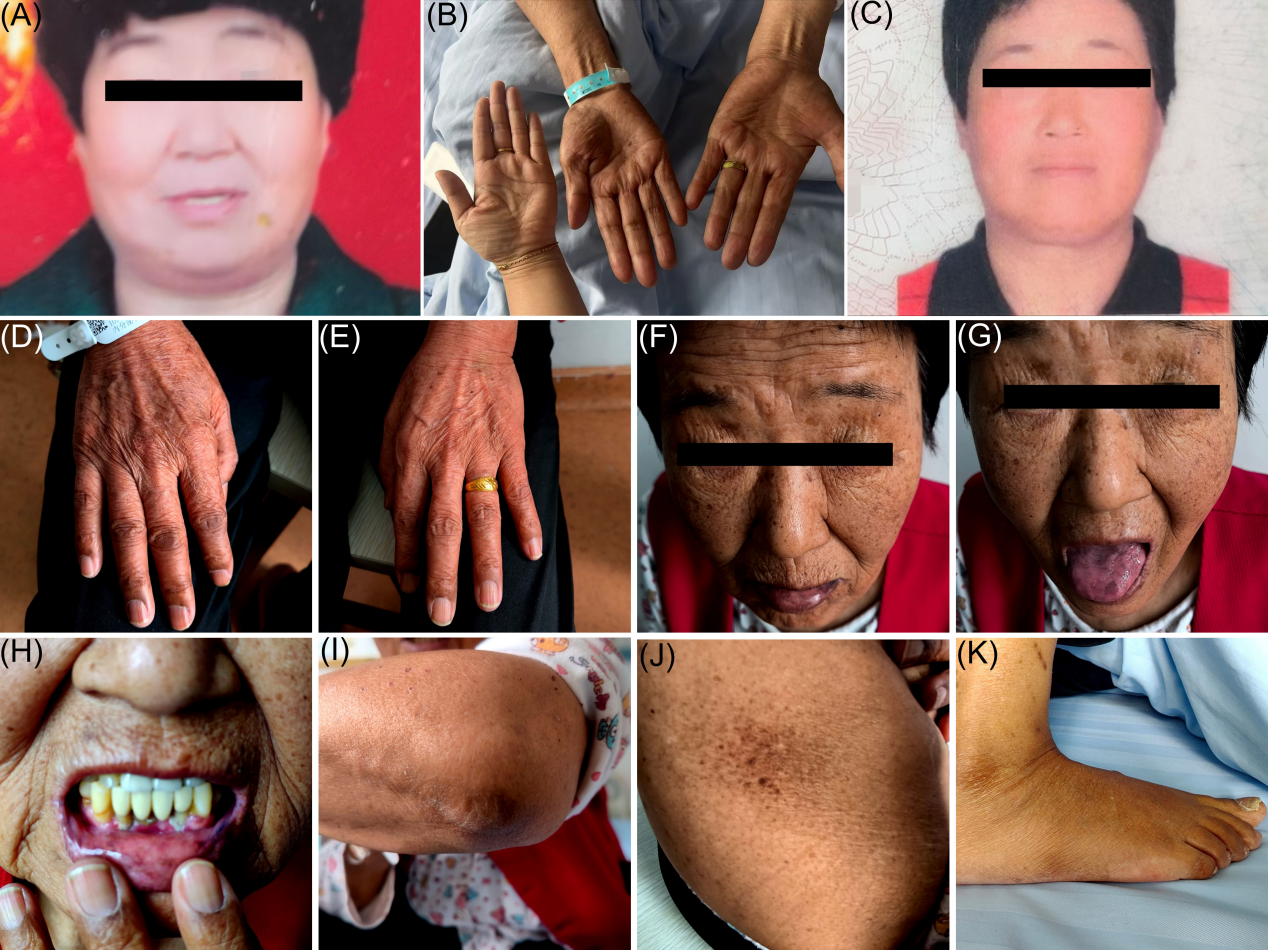
Body part images at different stages. **(A)** shows a facial image of the patient before illness onset; **(B)** shows an image of the patient's hand palm color and, for comparison, the treating physician’s hand palm color at the time of admission; **(C)** shows a facial image of the patient after treatment; **(D–K)** show images of the patient's skin pigmentation on the back of the hands, face (lips, tongue, gums), elbows, buttocks, and feet, at the time of admission.

**Figure 4:**
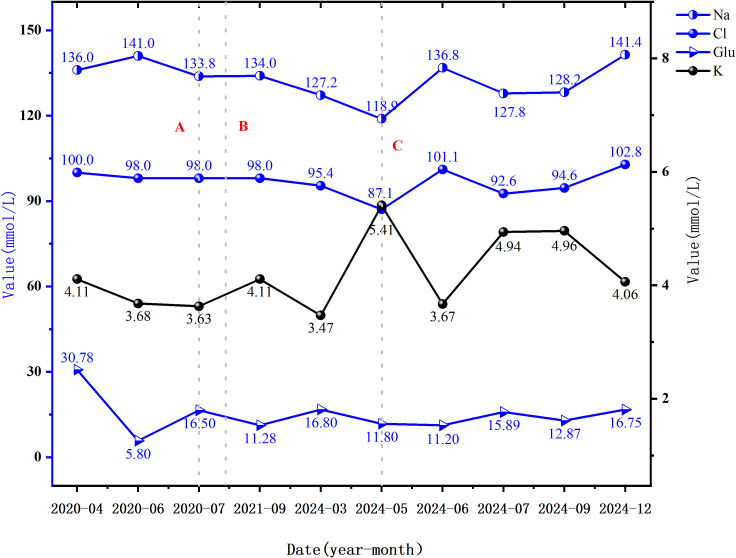
Blood glucose and electrolyte levels at various time points. Dashed line A represents the time of the first onset of hyponatremia; Dashed line B indicates the time of the first onset of skin and mucosal hyperpigmentation; Dashed line C marks the time of the first onset of hypochloremia (i.e., the time of the current admission). Abbreviations: Na, Sodium (135-145 mmol/L); Cl, Chloride (99-110 mmol/L); K, Potassium (3.5-5.5 mmol/L); Glu, Fasting glucose (3.90-6.10 mmol/L).

During follow-up, fatigue and gastrointestinal symptoms fully resolved, and skin pigmentation almost completely cleared. Persistent mild hyponatremia prompted the initiation of fludrocortisone acetate (0.05 mg/day) to address suspected adrenal zona glomerulosa dysfunction. At the final follow-up, laboratory and clinical parameters remained normal, with no further episodes of decompensation (**Figures 1**–**4**; **Table 1**).

**Table 1:** Patient laboratory and hormonal test results.

Analyte	Result	Reference interval	Unit
Fasting Blood Glucose	20.5 ↑	3.9~6.1	mmol/L
Glycated Hemoglobin	9.2% ↑	4.0~6.0	%
Fasting Insulin	0.54 ↓	3.00~25.00	mIU/L
Fasting C-Peptide	0.17 ↓	0.48~5.05	ng/mL
2-Hour Postprandial Insulin	55.71 ↑	3.00~25.00	mIU/L
2-Hour Postprandial C-Peptide	0.12 ↓	0.48~5.05	ng/mL
Insulin Autoantibody	16.40	0.00~20.00	IU/mL
Anti-Glutamic Acid Decarboxylase Antibody	102.00 ↑	0.00~30.00	IU/mL
Antinuclear Antibody	Positive	Negative	–
Anti-double stranded DNA antibody	Negative	Negative	–
Complement component 3	1.2	0.7~1.4	g/L
Complement component 3	0.25	0.10~0.40	g/L
Serum Creatinine	56	41~81	μmolL
pH Value	7.34↓	7.35~7.45	–
Base Excess	-4.8 ↓	-3.0~3.0	mmol/L
Urine Glucose	Positive(1+)	Negative	–
Urine Ketone Bodies	Positive (2+)	Negative	–
24-Hour Urine Sodium Excretion	358 ↑	130-260	mmol/L
Urine Protein	Negative	Negative	–
Luteinizing Hormone	21.23	15.90~54.00	mIU/mL
Follicle-Stimulating Hormone	44.67	23.00~116.30	mIU/mL
Growth Hormone	8.37 ↑	0.00~5.00	ng/mL
Prolactin	80.78 ↑	1.80~20.30	ng/mL
Insulin-like Growth Factor-1	16.90 ↓	31.00~323.00	ng/mL
Parathyroid Hormone	14.00	11.00~67.00	pg/mL
White Blood Cell Count	9.8 ↑	3.5-9.5	×10^9^/L
C-Reactive Protein	10.59 ↑	<10.00	mg/L
N-terminal pro-B-type Natriuretic Peptide	22863.00 ↑	≤125.00	pg/mL
High-Sensitivity Troponin T	33.05 ↑	≤14.00	ng/L
Adrenocorticotropic hormone_ at admission	>1250.00 ↑	5.00~46.00	pg/mL
Cortisol_ at admission	2.38 ↓	5.00~25.00	μg/dL
Cortisol, 60 min after cosyntropin (1 μg)	1.8 ↓	>18.0	μg/dL
Adrenocorticotropic hormone_ at discharge	1237.00↑	5.00~46.00	pg/mL
Cortisol_ at discharge	1.62↓	5.00~25.00	μg/dL
Adrenocorticotropic hormone_ 1-month post-discharge	589.00↑	5.00~46.00	pg/mL
Cortisol_ 1-month post-discharge	1.62↓	5.00~25.00	μg/dL
Adrenocorticotropic hormone_ 3 months post-discharge	55.30↑	5.00~46.00	pg/mL
Cortisol_ 3 months post-discharge	5.06	5.00~25.00	μg/dL
Adrenocorticotropic hormone_ 6 months post-discharge	19.90	5.00~46.00	pg/mL
Cortisol_6 months post-discharge	5.16	5.00~25.00	μg/dL

“-” means that the corresponding indicator has no unit.

"↑" means higher than the reference range and "↓" means lower than the reference range.

## 3 Discussion

### 3.1 Characteristics of APS-2

Autoimmune polyendocrine syndrome (APS) encompasses a group of autoimmune disorders (AIDs) involving two or more endocrine glands or non-endocrine organs. It is characterized by organ-specific lymphocytic infiltration and progressive functional failure mediated by circulating autoantibodies (1). APS is classified into three major subtypes based on genetic patterns and clinical features: APS-1 (associated with *AIRE* gene mutations), APS-2, and X-linked immune dysregulation syndrome. APS-2, also known as Schmidt syndrome and first described in 1926, is the most common subtype. It primarily affects the adrenal glands, thyroid, and pancreas, and it may coexist with non-endocrine AIDs such as myasthenia gravis (MG) (11). APS-2 is a polygenic disorder, involving multiple genetic loci and environmental factors, leading to significant heterogeneity in organ-specific damage. Major histocompatibility complex (*MHC*) genes on chromosome 6 are implicated in its pathology (12). Epidemiological studies estimate the prevalence of APS-2 at approximately 1/20,000, with a female-to-male ratio of 1.8–4.0:1 and peak onset occurring between 20 and 40 years of age (5). Notably, the full triad of adrenal, thyroid, and pancreatic involvement is extremely rare, with only four cases previously reported that progressed to AC. The present case represents the fifth reported instance of the full APS-2 triad complicated by AC. The patient’s prolonged diagnostic journey highlights the clinical challenges posed by the asynchronous progression of multi-glandular involvement, nonspecific symptoms, and insufficient clinical awareness.

### 3.2 Cascade mechanisms from single-gland damage to APS-2

This patient’s progressive disease offers a unique perspective into the pathophysiological mechanisms underlying the transformation from single-gland AID to full APS-2, involving the following key processes.

#### 3.2.1 Genetic susceptibility

APS-2 develops in individuals with polygenic susceptibility strongly associated with specific HLA haplotypes (e.g., *HLA-DR3/DR4*). These genes may disrupt antigen presentation or immune tolerance mechanisms, increasing susceptibility to multi-organ autoimmunity (13, 14). For instance, *HLA-DQ* and *HLA-DR* alleles may predispose to abnormal immune responses targeting thyroid, pancreatic β-cell, or adrenal cortical antigens. Polymorphisms in immune regulatory genes, such as *CTLA-4* (rs3087243) and *PTPN22* (rs2476601), can also impair T-cell receptor signaling and regulatory T-cell (Treg) function, exacerbating autoimmune progression (14). These genetic variations create a permissive microenvironment for cross-reactive immune responses against organ-specific antigens such as thyroglobulin, insulin, and 21-hydroxylase (15). Although genetic testing was not performed in this patient, the coexistence of thymoma and multiglandular involvement suggests the possible presence of *HLA-DR3/DR4* haplotypes or other immune regulatory gene variants.

Notably, the progression of PAI in this patient was unrelated to treatments for thymoma-associated MG. First, thymoma resection occurred prior to the onset of hyponatremia. Second, perioperative glucocorticoids were only briefly administered, with no prolonged immunosuppressants or cholinesterase inhibitors post-surgery being provided. Thus, the association between APS-2 and thymoma likely reflects shared autoimmune susceptibility rather than iatrogenic effects.

#### 3.2.2 Immune dysregulation

The central mechanism of APS-2 involves a progressive breakdown of immune tolerance. A reduction in Treg numbers (decreased CD4+CD25+FoxP3+ cell proportions) and impaired Treg function (reduced IL-10/TGF-β secretion) lead to uncontrolled activation and proliferation of autoreactive T cells (16). For example, autoreactive T cells in PAI target 21-hydroxylase, while similar Th1/Th17-polarized responses may extend via chemokine networks to thyroid follicular cells and pancreatic β cells, triggering Graves’ disease or T1DM (14, 17). In this case, thymectomy, critical for T-cell development, may have permitted the escape of autoreactive T-cells, and the absence of postoperative immunosuppression potentially accelerated the cascade-like autoimmune process.

#### 3.2.3 Antigen spread and molecular mimicry

Single-gland autoimmune damage can lead to multi-organ involvement through epitope spreading. For example, thyroid follicular destruction releases intracellular antigens such as thyroglobulin, which are captured by dendritic cells and cross-presented in lymph nodes, activating T-cell clones that subsequently target adrenal or pancreatic tissues (18). Environmental triggers (e.g., enteroviruses) may also disrupt immune tolerance via molecular mimicry (19). For instance, the PEVKEK sequence in Coxsackievirus B4 2C protein shares homology with the GAD65 antigen, potentially initiating cross-reactive immune responses against pancreatic islets (20). Similarly, *Mycobacterium avium* MAP3865c protein shares epitopes with pancreatic zinc transporter 8 (ZnT8) (21). The acute exacerbation of PAI symptoms after exposure to TCM in this case suggests that exogenous agents might promote antigen spreading via similar mechanisms.

#### 3.2.4 Cumulative effects of autoantibodies

Autoantibodies associated with single-gland AID may predict the risk of subsequent multi-gland involvement. For example, patients with 21-hydroxylase antibody (21-OHAb)-positive PAI have approximately a 50% risk of developing additional endocrine autoimmune disorders, suggesting that antibodies may contribute to tissue damage via complement activation or antibody-dependent cellular cytotoxicity (13). Notably, certain antibodies (e.g., anti-interferon-α) may also compromise innate immunity, creating a vicious cycle of infection and autoimmunity (22). In this patient, the progressive expansion of autoantibodies (IAA → Anti-GAD → TPOAb/TGAb) illustrates this cumulative autoimmune damage. Importantly, the patient’s ANA positivity—which are commonly associated with systemic lupus erythematosus, Sjögren’s syndrome, and systemic sclerosis—warrants regular monitoring of ANA titers and prompt evaluation for symptoms such as facial rash, recurrent oral ulcers, severe alopecia, polyarthralgia, dry mouth or eyes, frothy urine, or unexplained fever (23, 24).

Based on these mechanisms, we propose a structured monitoring approach for patients with single-gland AID (25, 26): 1) initial comprehensive screening for relevant autoantibodies (e.g., 21-OHAb, TPOAb, GAD65Ab); 2) biannual or annual evaluation of target organ function (morning cortisol, thyroid function, oral glucose tolerance test); and 3) prompt assessment for new glandular involvement when nonspecific symptoms (e.g., fatigue, weight loss) arise.

### 3.3 Diagnosis and differential diagnosis of APS-2

The patient presented with chronic fatigue, hyponatremia, orthostatic hypotension, and mucocutaneous hyperpigmentation. Prominent clinical features in admission included nausea, vomiting, severe anorexia, and hypovolemic shock. Laboratory findings indicated low cortisol and markedly elevated ACTH, consistent with PAI (27). Differential diagnoses were systematically considered and ultimately excluded: 1) Secondary adrenal insufficiency (SAI): Elevated ACTH levels and a normal pituitary CT effectively ruled out SAI (28); 2) Chronic kidney disease (CKD)-related electrolyte disturbances: Normal serum creatinine, eGFR, and absence of proteinuria excluded CKD as a cause of hyponatremia (29); 3) Myxedema: Though the patient had a history of primary hypothyroidism, stable TSH levels under adequate levothyroxine replacement therapy excluded thyroid-related etiology. Hypothyroidism-associated hyponatremia typically involves antidiuretic hormone (ADH) dysregulation and impaired free water excretion, whereas this patient exhibited hyponatremia with elevated urinary sodium excretion inconsistent with renal water retention mechanisms (30); 4) Gastrointestinal disorders: Normal gastrointestinal imaging and absence of endoscopic evidence excluded these diagnoses (31); 5) Malignancy/paraneoplastic syndrome: No unexplained fever, acute weight loss (4 kg loss correlated with acute decompensation), normal tumor markers (CEA, CA199), and absence of masses on thoracic/abdominal CT (prior thymoma resection without recurrence) ruled out this possibility (32); 6) Syndrome of inappropriate antidiuretic hormone secretion (SIADH): SIADH typically presents with euvolemic or mildly hypervolemic hyponatremia with normal cortisol/ACTH levels (33). The presence of severe hyponatremia with elevated urinary sodium and hypovolemia supported renal sodium wasting characteristic of PAI (34). The ACTH stimulation test further confirmed primary adrenal cortex dysfunction.

Following confirmation of PAI, etiological evaluation was conducted. Major causes of PAI include tuberculosis, autoimmune disorders, infections, tumors, and medications. This patient had no history of tuberculosis, systemic fungal infections, or HIV. Adrenal CT revealed no structural abnormalities, excluding adrenalectomy, hemorrhage, tumors, infiltration, congenital adrenal hyperplasia, or infectious adrenalitis (35, 36). The absence of long-term use of adrenal enzyme inhibitors (e.g., ketoconazole) ruled out drug-induced PAI (36). Adrenoleukodystrophy, typically affecting prepubertal males, was unlikely given the patient’s adult-onset presentation. The patient’s clinical history of MG and multiple autoimmune antibodies strongly suggested immune dysregulation, supporting autoimmune PAI (35). APS-2 diagnostic criteria require the coexistence of at least two endocrine AIDs (AITD, PAI, T1DM) with corresponding autoantibodies (5). This patient met these criteria based on her sequential diagnoses of T1DM, AITD, and autoimmune PAI. Although adrenal-specific antibodies were not tested, the patient’s autoimmune background and thorough exclusion of alternative causes confirmed APS-2.

Diagnostic challenges in this case included: 1) Nonspecific symptoms and overlapping clinical conditions: Chronic fatigue and anorexia were attributed initially to concurrent conditions such as MG-related muscle weakness, AITD-induced metabolic slowdown, and catabolic changes from DKA. The insidious onset and nonspecific nature of PAI, compounded by limited clinician awareness, contributed to delays in the recognition of key indicators such as hyponatremia, hyperpigmentation, and orthostatic hypotension. 2) Complex pathophysiology of hyponatremia: Persistent hyponatremia necessitated a differentiation between hypothyroidism and DKA. In hypothyroidism, hyponatremia arises from impaired water excretion, abnormal ADH secretion, aquaporin dysregulation, and reduced cardiac output (37–40). DKA-related hyponatremia results primarily from dehydration and osmotic diuresis (41, 42). Conversely, in PAI, hyponatremia results from combined aldosterone and cortisol deficiencies, leading to sodium wasting, water retention, and dysregulated ADH secretion (43, 44). Although treatment with 10% sodium chloride temporarily normalized serum sodium levels during hospitalization, persistent hyponatremia post-discharge indicated involvement of the adrenal zona glomerulosa. This dynamic course highlights the importance of a careful evaluation of the multifactorial nature of hyponatremia in polyendocrine disorders to avoid misdiagnosis.

### 3.4 Diagnosis and differential diagnosis of AC

AC represents the most severe manifestation of PAI, occurring in patients with APS-2 when cortisol production becomes inadequate or cortisol requirements increase due to acute stressors such as infection, surgery, excessive sweating, vomiting, or inadequate glucocorticoid treatment (6). Without prompt glucocorticoid replacement, AC rapidly becomes life-threatening, with an annual incidence of 6%–8% (6). AC is diagnosed when a patient with PAI experiences acute deterioration with at least two of the following symptoms or signs: hypotension, acute abdominal pain, nausea/vomiting, altered mental status, fatigue, fever, or biochemical abnormalities such as hyponatremia, hyperkalemia, or hypoglycemia, necessitating immediate intravenous glucocorticoid administration (6). This patient presented with an acute exacerbation of fatigue, nausea/vomiting, severe hyponatremia, reduced insulin requirements, and evidence of hypovolemic shock. Intravenous sodium hydrocortisone succinate (100 mg Q8h) was promptly initiated. This led to a rapid clinical improvement within 24 hours, including resolution of fatigue, improved appetite, and normalization of vital signs, confirming the diagnosis of AC.

A critical differential diagnosis to consider is stress-induced cardiomyopathy (Takotsubo syndrome, TTS, characterized by transient, reversible left ventricular dysfunction often triggered by intense emotional or physical stress such as emotional trauma, surgery, or acute illness. The pathophysiology of TTS involves excessive catecholamine release, causing microvascular dysfunction and myocardial stunning (45). Under normal conditions, cortisol mitigates catecholamine-induced endothelial damage by suppressing inflammatory cytokines (e.g., IL-6, TNF-α) and modulating the hypothalamic-pituitary-adrenal (HPA) axis to prevent sympathetic hyperactivation. In cortisol states of cortisol deficiency such as PAI, compensatory sympathetic hyperactivation and catecholamine excess (46, 47), combined with uncontrolled inflammation and increased β-receptor sensitivity amplify cardiotoxicity (48–50), resulting in microvascular spasms, calcium overload, oxidative stress, and excitation-contraction uncoupling (51–54). In patients with PAI, TTS can manifest as acute left heart failure, cardiogenic shock, or refractory arrhythmias (e.g., torsades de pointes), with echocardiography showing characteristic regional wall motion abnormalities. A previously reported case described TTS with cardiogenic shock in a patient with PAI following corticotropin-releasing hormone (CRH) testing, highlighting iatrogenic stress as a potential trigger (55). Chronic under-treatment with glucocorticoids increases TTS risk during periods of infection or surgery (56, 57).

Although the patient described in this report presented initially with features suggestive of acute left heart failure and cardiogenic shock, initial electrocardiography and echocardiography revealed no abnormalities, precluding the need for further serial cardiac ultrasound monitoring. Nonetheless, clinicians should remain vigilant for TTS in AC patients presenting with acute cardiac symptoms or unexpected cardiac deterioration during glucocorticoid therapy or stress events. Optimizing glucocorticoid dosing reduces catecholamine-related toxicity risk, while prompt echocardiography facilitated the early detection of TTS (57).

### 3.5 Multiple mechanisms inducing AC

In contrast with previously reported APS-2 cases, this patient’s AC involved multifactorial triggers, including thyroid hormone replacement therapy, DKA, acute heart failure, and potential TCM-induced adrenal toxicity. This case highlights the cumulative effects of levothyroxine replacement and TCM exposure in accelerating the progression of PAI.

The regulatory effects of thyroxine on adrenal cortical function may involve four mechanisms: 1) Accelerated cortisol clearance: Levothyroxine increases basal metabolic rate and induces hepatic cytochrome P450 enzymes (e.g., CYP3A4), enhancing cortisol hydroxylation and conjugation, thereby accelerating conversion to inactive metabolites such as cortisone (58, 59). Additionally, thyroid hormones may modulate 11β-hydroxysteroid dehydrogenase activity, influencing cortisol-cortisone interconversion (60, 61). 2) HPA axis dysregulation: Short-term levothyroxine therapy enhances CRH/ACTH secretion to stimulate cortisol production. However, chronic treatment may suppress TSH via negative feedback, potentially reducing adrenal reserve function (58, 62, 63). 3) Receptor-level cross-regulation: Co-expression of thyroid hormone receptors (TRs) and glucocorticoid receptors (GRs) can lead to receptor cross-talk. Low cortisol levels observed in AITD patients may stem from TR-mediated upregulation of GR sensitivity, accelerating cortisol catabolism (64–66). 4) Direct inhibition of steroid synthesis: Animal studies indicate that exogenous thyroxine downregulates steroidogenic acute regulatory protein (StAR) and cholesterol side-chain cleavage enzyme (CYP11A1) in adrenal cortical cells, impairing critical steps in cortisol synthesis and reducing adrenal responsiveness to ACTH (62, 67–69). Clinicians should therefore suspect PAI in hypothyroid patients who present with persistent fatigue despite adequate thyroid replacement therapy (26). Further research is needed to clarify how thyroxine differentially affects cortisol metabolism across various pathological states (62, 65, 70).

Additionally, this case provides clinical evidence, through a clear temporal association, that TCM exposure may accelerate APS-2 progression. Potential mechanisms include: 1) Mineralocorticoid-like effects: Glycyrrhizin derivatives inhibit 11β-hydroxysteroid dehydrogenase type 2 (11β-HSD2), leading to abnormal renal cortisol accumulation, mimicking mineralocorticoid activity, and subsequently suppressing the renin-angiotensin-aldosterone (RAA) system (71–74). Chronic exposure may lead to zona fasciculata atrophy and compensatory ACTH elevation (75, 76). 2) Disruption of glucocorticoid metabolism: Components such as tanshinone IIA and triptolide induce CYP3A4 expression by activating nuclear receptors (e.g., PXR) and epigenetic modifications (e.g., H3K4me2 methylation), thus accelerating glucocorticoid metabolism (77–80). Although specific TCM components responsible for the observed clinical deterioration in this case remain unidentified, the clear temporal correlation between TCM use and the clinical manifestations (e.g., worsening fatigue, hyperpigmentation) supports a causal relationship between TCM exposure and progression from subclinical to overt PAI. Clinicians should thoroughly assess the use of herbal products and dietary supplements in APS-2 patients and avoid prolonged administration of agents potentially disruptive to the adrenal axis- without robust pharmacological evidence (81).

### 3.6 Treatment strategies, prognosis, and limitations

Physiologic-dose hormone replacement remains the cornerstone of APS-2 management. Given the interconnected nature of endocrine gland dysfunction, treatment necessitates coordinated and timely dose adjustments (26). In this case, electrolyte and metabolic abnormalities were effectively corrected with adequate hormonal combination therapy. At the last follow-up, laboratory parameters including serum sodium (141.4 mmol/L), potassium (4.06 mmol/L), basal cortisol (5.72 μg/dL), and ACTH (19.9 pg/mL) had normalized, accompanied by a complete resolution of fatigue and anorexia. A comprehensive three-tier management strategy was implemented, emphasizing patient education: 1) Daily monitoring of blood glucose, blood pressure, and symptom diaries; 2) Stress response measures, such as doubling glucocorticoid doses during infection or/trauma; 3) Emergency preparedness, including carrying an emergency card detailing the patient’s diagnosis and medication regimen.

Study limitations: The presented case focused primarily on evaluating cortisol deficiency during hospitalization. The absence of serum potassium abnormalities led to an incomplete assessment of the RAA system, highlighting the importance of a systematic evaluation of this axis in PAI patients, even when potassium levels are found to be normal. Emerging evidence indicates that approximately 35% of autoimmune PAI patients maintain normal serum potassium during early zona glomerulosa dysfunction. Additionally, laboratory constraints precluded testing for anti-adrenal cortex antibodies and acetylcholine receptor antibodies. Despite these limitations, systematic clinical evaluation effectively excluded common secondary PAI causes, including infections, metabolic causes, or iatrogenic factors. Furthermore, this case highlights that negative 21-OHAb results do not fully exclude autoimmune PAI, particularly in patients with advanced disease or coexisting autoimmune conditions, where antibody-negative rates can reach 30% (82). Comprehensive diagnosis in antibody-negative cases should integrate dynamic antibody monitoring, adrenal function assessments, and genetic testing to prevent missed diagnoses of autoimmune PAI (83).

### 3.7 Clinical features and literature analysis of APS-2 complicated by AC

AC is a leading cause of mortality among APS-2 patients, yet it remains poorly characterized. To improve clinical understanding, we analyzed previously reported APS-2 cases complicated by AC. A systematic literature review was conducted in PubMed (up to June 2024) using the search terms: *(“Polyendocrinopathies, Autoimmune”[Mesh] OR “autoimmune polyendocrine syndrome type 2 [Title/Abstract]”) AND (“Adrenal Insufficiency”[MeSH] OR “Addison Disease”[MeSH] OR “Adrenal Crisis”[Title/Abstract])*. Case reports and series meeting APS-2 diagnostic criteria with confirmed AC events were included, yielding 18 patients (**Table 2**).

**Table 2:** Overview of published cases involving APS-2 complicated by AC.

Author	Gender	Age (y)	Disease Composition	Initial Disease	Clinical Presentation	Trigger	Time^a^ (days)	Auto-Ab	Hyponatremia	Hyperkalemia	Hypovolemic Shock	Treatment	Prognosis
Nelson (2)	Female	11	T1DM+AITD+PAI	T1DM	weakness, anorexia, vomiting, fever	**-**	2920	TGAb (+) 21-OHAb (+)	Yes	Yes	Yes	prednisolone + LT4 + fludrocortisone + insulin	growth retardation
Murray (84)	Female	26	AITD+PAI	T1DM	lethargy, vomiting, nausea, hypoglycemia	LT4-therapy	150	TPOAb (+) 21-OHAb (+)	Yes	Yes	Yes	glucocorticoids+ fludrocortisone +LT4	clinical stability
Mazul-Sunko (85)	Female	43	AITD+PAI	PAI	weakness, vomiting, fatigue	Surgery	0	TPOAb (+) 21-OHAb (+)	No	No	Yes	hydrocortisone + LT4	clinical stability
Tsang (3)	Female	42	T1DM+AITD+PAI	AITD	weakness, nausea, vomiting	Infection	1275	TPOAb (+) TGAb (+)	Yes	Yes	Yes	hydrocortisone + LT4+ insulin	clinical stability
Ghanny (86)	Male	6	T1DM +PAI	T1DM	lethargy, vomiting, hyperpigmentation	**-**	2005	21- OHAb (+)	Yes	**-**	**-**	hydrocortisone + fludrocortisone + insulin	clinical stability
Chang (87)	Male	4	T1DM +PAI	T1DM	hypoglycemia, hyperpigmentation	**-**	180	–	Yes	No	Yes	hydrocortisone + fludrocortisone + insulin	clinical stability
Vallianou (88)	Female	74	AITD+PAI	AITD	fatigue, nausea, vomiting, hyperpigmentation	LT4-therapy	5	TPOAb (+) TGAb (+) 21-OHAb (+)	Yes	Yes	Yes	adrenal steroid hormone+ LT4	–
Bain (89)	Female	57	AITD+PAI	AITD	weight and appetite loss, vomiting, hyperpigmentation	Infection	3285	TPOAb (+) TGAb (+) 21-OHAb (+)	Yes	No	Yes	hydrocortisone + fludrocortisone+ LT4	clinical stability
Gürkan (90)	Female	36	AITD+PAI	–	nausea, vomiting, appetite loss	Shock	0	TPOAb (+) TGAb (+)	Yes	Yes	Yes	methyl prednisolone+ fludrocortisone	clinical stability
Wang (91)	Female	38	AITD+PAI	AITD	hyperpigmentation, appetite loss, nausea	**-**	850	TPOAb (+) TGAb (+)	Yes	Yes	Yes	hydrocortisone + fludrocortisone+ LT4	clinical stability
Schulz (92)	Male	15	AITD+PAI	GD	weight loss, nausea, vomiting, hyperpigmentation	**-**	180	TPOAb (+) TRAb (+) 21-OHAb (+)	Yes	No	Yes	hydrocortisone + fludrocortisone + carbimazole	clinical stability
Yanachkova (93)	Female	41	AITD+PAI	AITD	hyperpigmentation	Pregnancy	5840	–	Yes	Yes	**-**	prednisolone + fludrocortisone + LT4	clinical stability
Wiśniewski (94)	Female	78	AITD+PAI	AITD	diarrhea, vomiting, weakness, fatigue	**-**	**-**	TPOAb (+) TGAb (+)	Yes	No	Yes	hydrocortisone + fludrocortisone+ LT4	clinical stability
Lassoued (95)	Female	28	AITD+PAI	AITD	fever, palpitation, vomiting, weight loss	Thyroid Crisis	0	TPOAb (+) TGAb (+)	Yes	Yes	Yes	hydrocortisone + LT4	clinical stability
Lantz (4)	Female	21	T1DM+AITD+PAI	–	nausea, vomiting, weakness	Infection	**-**	–	Yes	Yes	Yes	LT4+insulin+hydrocortisone+ fludrocortisone	clinical stability
Bonataki (96)	Female	11	AITD+PAI	PAI	hyperpigmentation, vomiting, weight loss, fatigue, weakness	–	2190	TPOAb (+), TGAb (+) 21-OHAb (+)	Yes	Yes	Yes	hydrocortisone+ fludrocortisone	clinical stability
Spagnolo (97)	Female	55	AITD+PAI	AITD	weight loss, nausea, vomiting, palpitation	ICPis	10	TPOAb (+) TGAb (+)	Yes	Yes	Yes	hydrocortisone + fludrocortisone+ LT4	clinical stability
Pan (5)	Female	60	T1DM+AITD+PAI	AITD	weight loss, nausea, vomiting, weakness	ICPis	150	TPOAb (+) TGAb (+)	Yes	Yes	No	hydrocortisone+ LT4+ insulin	occasional hypoglycemia
This case	Female	69	T1DM+AITD+PAI	T1DM	Fatigue, appetite loss, weight loss, nausea, vomiting, weakness, hyperpigmentation	LT4 therapy +DKA+ AHF+TCM	3285	TPOAb (+), TGAb (+) IAA (+), ANA (+) GAD65Ab (+)	Yes	No	Yes	prednisolone + fludrocortisone + LT4+insulin	clinical stability

Time^a^ (days), days ever since the initial disease to the diagnosis of APS-2; AC, Adrenal crisis; ICPis, immune checkpoint inhibitors; T1DM, type 1 diabetes mellitus; PAI, autoimmune adrenal insufficiency; AITD, autoimmune thyroid disease; TPOAb, thyroid peroxidase antibody; TGAb, thyroglobulin antibody; TRAb, thyroid-stimulating hormone receptor antibody; 21-OHAb, 21-hydroxylase antibody; IAA, insulin autoantibody; ANA, antinuclear antibody; GAD65Ab, anti-glutamic acid decarboxylase 65 antibody; LT4, levothyroxine; DKA, Diabetic ketoacidosis; AHF, Acute heart failure; TCM, traditional Chinese medicine; USA, United States of America; UK, United Kingdom. "-" means not mentioned in the article.

#### 3.7.1 Case characteristics

Demographics and Geographic Distribution: Females predominated (15/18, 83.3%; male-to-female ratio 1:5). The age of onset ranged widely (4–74 years; mean 35.5 ± 22.5 years), with the highest proportion (44.4%) aged >40 years. Geographically, China (22.2%) and the U.S. (16.7%) contributed the most cases.

Initial Clinical Encounters and Diagnostic Delays: 46.7% (7/15) of patients initially presented to emergency departments, while only 13.3% (2/15) sought care at endocrinology clinics, likely reflecting the acute nature of AC, typically manifesting with hypovolemic shock (93.8%). The median time from first disease onset to APS-2 diagnosis was 180 days (range: 0–5840 days). In the presented case, the patient experienced a markedly prolonged diagnostic delay of 3,285 days, far exceeding the median, underscoring the need for a heightened awareness and systematic adrenal function evaluations in patients presenting with persistent fatigue, hyponatremia, and orthostatic hypotension, particularly in the presence of concurrent T1DM or AITD.

#### 3.7.2 Crisis triggers and disease patterns

Triggers: Specific precipitating factors were identified in 61.1% (11/18) of AC cases, most commonly these included infections (27.3%), levothyroxine replacement (18.2%), and immune checkpoint inhibitors (18.2%). Our case uniquely highlights AC triggered by multiple concurrent factors, including levothyroxine replacement, DKA, acute heart failure, and TCM exposure, suggesting that cumulative stressors may elevate the risk of adrenal decompensation (26).

Disease Combinations: All patients exhibited PAI, 88.9% had AITD, and 38.9% had T1DM. The most common disease combination was AITD+PAI (66.7%), whereas the complete triad (T1DM+AITD+PAI) accounted for only 22.2% of reported cases. Our study contributes the fifth globally reported case of full-triad APS-2 complicated by AC, further expanding the spectrum of clinical disease manifestations. Initial presentations included AITD (55.6%, 10/18), T1DM (22.2%, 4/18), and PAI (11.1%, 2/18), with only one case exhibiting simultaneous onset of all three conditions.

#### 3.7.3 Biochemical features

Hyponatremia occurred in 94.4% (17/18) of cases and hyperkalemia in 70.6% (12/17). In our case, chronic anorexia and vomiting likely masked significant potassium elevation, highlighting the importance of evaluating nutritional status when interpreting electrolyte abnormalities to avoid misdiagnosis.

### 3.8 Early identification and long-term management.

Autoimmune PAI typically develops insidiously, with immune dysregulation typically preceding overt PAI by several years. The natural disease progression includes five-stages and can be dynamically assessed using the ACTH stimulation test (98, 99). This test evaluates baseline cortisol, ACTH, upright renin, aldosterone, and cortisol responses at 60 minutes post-ACTH administration. These parameters establish diagnostic stages as follows: Stage 0 (normal adrenal function), Stage I (elevated renin with normal/low aldosterone, indicating early adrenal cortical dysfunction), Stage II (markedly elevated renin, reduced aldosterone, and abnormal cortisol response), Stage III (elevated ACTH with normal/low cortisol), and Stage IV (significant ACTH elevation, low cortisol, and classic PAI symptoms). Typically, zona glomerulosa dysfunction (aldosterone production) precedes impairment of the zona fasciculata and reticularis (cortisol synthesis) due to local cortisol protection. For patients positive for 21-OHAb, periodic ACTH stimulation tests combined with RAA monitoring are recommended, enabling the timely initiation of hormone replacement therapy at subclinical stages (Stage I) to delay overt PAI.

Long-term APS-2 management requires multidisciplinary collaboration and structured follow-up. Annual screening should encompass thyroid function (TSH, FT4, TPOAb, TGAb), adrenal function (morning cortisol, ACTH, 21-OHAb), gonadal function (FSH/estradiol in females, testosterone in males), and glucose metabolism (HbA_1_c, C-peptide) (26, 100). Pregnancy necessitates more intensive monitoring of thyroid, glucose, and adrenal parameters (100–104). Screening for non-endocrine autoimmune complications such as pernicious anemia, autoimmune gastritis, and celiac disease, should include complete blood count, gastric parietal cell antibodies, intrinsic factor antibodies, and tissue transglutaminase antibodies (100–102). Asymptomatic antibody-positive individuals should undergo evaluations every 3–6-months, and regular screening is also advised for first-degree relatives (26). Clinicians should systematically document symptoms such as fatigue, weight changes, hyperpigmentation, thyroid enlargement, and vitiligo as part of a comprehensive assessment (101–103, 105).

Emerging diagnostic tools include combined antibody panels (e.g., IL-17/IL-22 autoantibodies alongside GAD65, 21-OHAb, TPOAb) (106), genetic risk profiling (e.g., HLA-DR3/DR4 haplotypes such as DRB1*0404) (107, 108), innovative functional assays (e.g., low-dose ACTH stimulation with dynamic renin activity) (109, 110), and omics technologies (e.g., metabolomics/proteomics to identify early metabolic abnormalities) (111). Current, APS-2 diagnosis remains primarily clinical and antibody-based. Future research efforts should focus on developing dynamic predictive models integrating antibody profiles, adrenal function, and genetic risk data, alongside AI-assisted diagnostic algorithms employing immunometabolic biomarkers for early detection and intervention.

## 4 Conclusion

This study reports the fifth case of full triad APS-2 complicated by AC. The multifactorial triggers of AC and prolonged diagnostic delay highlight key clinical challenges in managing APS-2: 1) nonspecific clinical presentations and asynchronous multiglandular involvement contributing to diagnostic delays; 2) the complexity of differentiating electrolyte disturbances such as hyponatremia, necessitating dynamic hormonal evaluations; and 3) the potential contribution of exogenous factors (e.g., TCM) in exacerbating adrenal insufficiency by disrupting cortisol metabolism and immune homeostasis. Consequently, we recommend: 1) stepwise monitoring though antibody screening and regular target organ function assessments in patients with single-gland autoimmune diseases; 2) vigilance for cortisol-ACTH axis dynamics during thyroid hormone replacement therapy; 3) cautious use of TCM to avoid potential adrenal toxicity; and 4) multidisciplinary management, including structured glucocorticoid adjustment during stress events for patients with an elevated risk for AC.

Study limitations include incomplete RAA axis evaluation and unavailability of specific antibody testing. Future research should integrate genetic and metabolomic information to refine predictive models, better understand TCM-related adrenal toxicity, and optimize early precision-based interventions. In summary, this case not only expands the clinical spectrum of APS-2 but also highlights the need for increased awareness by clinicians, systematic follow-up, and targeted preventive strategies to improve patient outcomes.

## Data Availability

The original contributions presented in the study are included in the article/supplementary material. Further inquiries can be directed to the corresponding author.
