# Corrigendum: Delayed diagnosis of the full triad autoimmune polyendocrine syndrome type 2 with adrenal crisis: a case report and literature review

**DOI:** 10.3389/fimmu.2025.1625708

**Published:** 2025-06-03

**Authors:** Zihong Yao, Hui Chen, Xuejian Hu, Dan Ge, Xiangyu Xu, Danxia Xu

**Affiliations:** ^1^ The Second Clinical Medical College, Lanzhou University, Lanzhou, Gansu, China; ^2^ Department of Endocrinology and Metabolism, Lanzhou University Second Hospital, Lanzhou, Gansu, China

**Keywords:** autoimmune polyendocrine syndrome type 2, adrenal insufficiency, adrenal crisis, hypothyroidism, diabetes mellitus

In the published article, there was an error in the author list, and authors “Chen Hui and Xu Danxia” were listed in the wrong order. The corrected author list appears below.

“Zihong Yao^1,2^, Hui Chen^2*^, Xuejian Hu^2^, Dan Ge^2^, Xiangyu Xu^2^ and Danxia Xu^2^”.

The authors apologize for this error and state that this does not change the scientific conclusions of the article in any way. The original article has been updated.

